# Mental health and well-being during the COVID-19 pandemic: longitudinal analyses of adults in the UK COVID-19 Mental Health & Wellbeing study

**DOI:** 10.1192/bjp.2020.212

**Published:** 2020-10-21

**Authors:** Rory C. O'Connor, Karen Wetherall, Seonaid Cleare, Heather McClelland, Ambrose J. Melson, Claire L. Niedzwiedz, Ronan E. O'Carroll, Daryl B. O'Connor, Steve Platt, Elizabeth Scowcroft, Billy Watson, Tiago Zortea, Eamonn Ferguson, Kathryn A. Robb

**Affiliations:** 1 Suicidal Behaviour Research Laboratory, Institute of Health & Wellbeing, University of Glasgow, UK; 2 Institute of Health & Wellbeing, University of Glasgow, UK; 3 Division of Psychology, University of Stirling, UK; 4 School of Psychology, University of Leeds, UK; 5 Usher Institute, University of Edinburgh, UK; 6 Samaritans, UK; 7 Scottish Association for Mental Health, UK; 8 Suicidal Behaviour Research Laboratory, Institute of Health & Wellbeing, University of Glasgow, UK; 9 School of Psychology, Nottingham University, UK

**Keywords:** COVID-19, mental health, suicidal ideation, general population, depression

## Abstract

**Background:**

The effects of coronavirus disease 2019 (COVID-19) on the population's mental health and well-being are likely to be profound and long lasting.

**Aims:**

To investigate the trajectory of mental health and well-being during the first 6 weeks of lockdown in adults in the UK.

**Method:**

A quota survey design and a sampling frame that permitted recruitment of a national sample was employed. Findings for waves 1 (31 March to 9 April 2020), 2 (10 April to 27 April 2020) and 3 (28 April to 11 May 2020) are reported here. A range of mental health factors was assessed: pre-existing mental health problems, suicide attempts and self-harm, suicidal ideation, depression, anxiety, defeat, entrapment, mental well-being and loneliness.

**Results:**

A total of 3077 adults in the UK completed the survey at wave 1. Suicidal ideation increased over time. Symptoms of anxiety, and levels of defeat and entrapment decreased across waves whereas levels of depressive symptoms did not change significantly. Positive well-being also increased. Levels of loneliness did not change significantly over waves. Subgroup analyses showed that women, young people (18–29 years), those from more socially disadvantaged backgrounds and those with pre-existing mental health problems have worse mental health outcomes during the pandemic across most factors.

**Conclusions:**

The mental health and well-being of the UK adult population appears to have been affected in the initial phase of the COVID-19 pandemic. The increasing rates of suicidal thoughts across waves, especially among young adults, are concerning.

## Background

The effects of coronavirus disease 2019 (COVID-19) on mental health and well-being are likely to be profound and long-lasting^1,2^ and will extend beyond those who have been directly affected by the virus. However, it is unclear who will be affected and to what extent such effects will generalise across all aspects of mental health.

Evidence from previous public health epidemics (for example the severe acute respiratory syndrome (SARS) virus) illustrated that the adverse effects are more common in some groups and that the detrimental effects are more pronounced among certain aspects of mental health than others.^3–7^ Increased risk of suicide was evident following SARS in older adults.^4^ Cross-sectional^8–10^ and longitudinal evidence (over 4 weeks)^11^ from China during the early stages of the outbreak of COVID-19 found high levels of mental health problems and distress in the general population. A study from Spain reported that distress during lockdown was associated with younger age and being female.^12^ Data from the University College London COVID-19 Social Study, which started post-pandemic, suggests self-harm and thoughts of suicide/self-harm were higher among women, Black, Asian and minority ethnic groups, people experiencing socioeconomic disadvantage and those with mental disorders.^13^ Repeated cross-sectional and longitudinal analysis of individual responses to the UK Household Longitudinal Study panel, including pre-pandemic data, have also demonstrated that mental health deteriorated in the early stages of the pandemic.^14^ All of these studies point to elevated rates of anxiety, depression, stress, suicide risk and post-traumatic stress in the initial stages of the pandemic.

On the 23 March 2020, a nationwide lockdown was announced by the UK government with the public instructed to stay at home, socially distance and self-isolate with strict guidance about movement outside of one's household. Public health measures are important to protect physical health, but it is essential that we gain a clearer understanding of the mental health and well-being of the UK population during the COVID-19 pandemic.^15^ Such understanding is vital to ensure that those affected receive the support that they require and to enable us to be better prepared for a potential second wave of the pandemic and for future outbreaks. Lockdown and the social and economic consequences of COVID-19 are likely to be associated with loneliness, social isolation and entrapment.^1^ To track their effects longitudinally, we assessed a wide range of mental health and well-being outcomes including: symptoms of depression and anxiety; well-being; defeat; entrapment; suicidal thoughts and behaviours; and loneliness.

## Aims

The current study investigates the mental health and well-being of adults in the UK in the early weeks of the COVID-19 pandemic. Using a quota survey design and a sampling frame that permitted recruitment of a national sample, we report the mental health and well-being of adults in the UK at three time points across 6 weeks following the COVID-19 lockdown in the UK. We also investigated whether outcomes varied by sociodemographic characteristics and those with pre-existing mental health problems, given their established vulnerability.^1,16,17^

## Method

### Study design, setting and participant recruitment

Participant recruitment was conducted by Taylor McKenzie, a social research company. We recruited a non-probability sample of adults (aged 18 years or older) from across the UK to the UK COVID-19 Mental Health & Wellbeing study (UK COVID-MH), with a longitudinal study design. We employed a quota sampling methodology, with quotas based on age (18–24 years: 12%; 25–34: 17%; 35–44: 18%; 45–54: 18%; 55–64: 15%; ≥65: 20%), gender (women: 51%; men: 49%), socioeconomic grouping (SEG; AB: 27%; C1: 28%; C2: 20%; DE: 25%, based on occupation, where A,B and C1 are  higher and categories C2, D, E are lower) and region of the UK (12 regions). The weighted and unweighted participant characteristics are presented in Table 1, with further details of gender identity and region reported in supplementary Tables 1 and 2. Weights are based on National Readership Survey and Office for National Statistics data for SEG and UK region, respectively and Census data for age and gender.^18,19^ Given the time sensitive nature of the study, a quota methodology was selected over probability sampling because it facilitated the recruitment of a well-stratified UK sample at the early phase of lockdown. Moreover, given the constraints of lockdown, online recruitment was the only feasible design.

**Table 1:** Demographic characteristics of the participants (*n* = 3077)

Characteristic	Total, not weighted: *n* (%)	Total, weighted: *n* (%)
Gender at birth^a^		
Men	1381 (44.9)	1470 (49.1)
Women	1692 (55.1)	1526 (50.9)
Ethnicity^b^		
White	2777 (90.5)	2691 (90.0)
Asian	162 (5.3)	169 (5.7)
Black	68 (2.2)	72 (2.4)
Mixed	52 (1.7)	48 (1.6)
Other	10 (0.3)	10 (0.3)
Relationship status		
Married/living with partner	1834 (59.6)	1790 (59.7)
Single	962 (31.3)	929 (31.0)
Separated/divorced/widowed	248 (8.1)	247 (8.2)
Other/prefer not to say	33 (1.1)	32 (1.1)
Sexuality		
Heterosexual	2830 (92.0)	2762 (92.1)
Gay or bisexual	220 (7.1)	212 (7.1)
Other/prefer not to say	27 (0.9)	26 (0.9)
Employment status		
Employed	1838 (59.7)	1806 (60.2)
Unemployed	358 (11.6)	342 (11.4)
Other (retired, education, homemaker)	881 (28.6)	852 (28.4)
Socioeconomic grouping^c^		
High	1758 (57.1)	1651 (55.0)
Low	1319 (42.9)	1349 (45.0)
Tenure		
Own (including with mortgage)	1835 (59.6)	1792 (59.7)
Private rent	694 (22.6)	682 (22.7)
Council rent	463 (15.0)	446 (14.9)
Other	85 (2.8)	81 (2.7)
Pre-existing mental health condition	836 (27.2)	780 (26.0)

a *n* = 3073.

b *n* = 3069.

c Categories A,B,C1, high socioeconomic group; categories C2, D, E low socioeconomic group.

Between 31 March and 9 April 2020, members of an existing online UK panel (Panelbase.net) were invited by email to take part in an online survey on health and well-being (wave 1). The panel has approximately 300 000 registered adult members. In total, 7471 panel members were invited to take part, 3077 were included in the final sample (target sample was *n* = 3000) and 4394 did not take part in the survey. The majority were screened out as a particular quota was full (*n* = 3527) and the remainder dropped out (*n* = 867; see Methods in the supplementary materials; available at https://doi.org/10.1192/bjp.2020.212). Respondents were asked demographic questions to determine whether they qualified for the study and if they did, they were re-directed to the survey.

After providing informed consent online, participants completed a wide range of psychological and social measures including questions about COVID-19. Only findings related to depression, anxiety, suicidal and self-harm history, defeat, entrapment, loneliness and well-being are reported here. Participants were informed that they would have the opportunity to participate in a minimum of six waves of the survey that would track the health and well-being of the UK during the current COVID-19 outbreak. All those who took part in wave 1 were invited to take part in the follow-up waves, and missing wave 2 did not exclude participation in wave 3. The follow-up surveys were scheduled to ensure a minimum of 1 week (wave 2) and 3 weeks (wave 3) between each participant completing a wave.

**Fig. 1:**
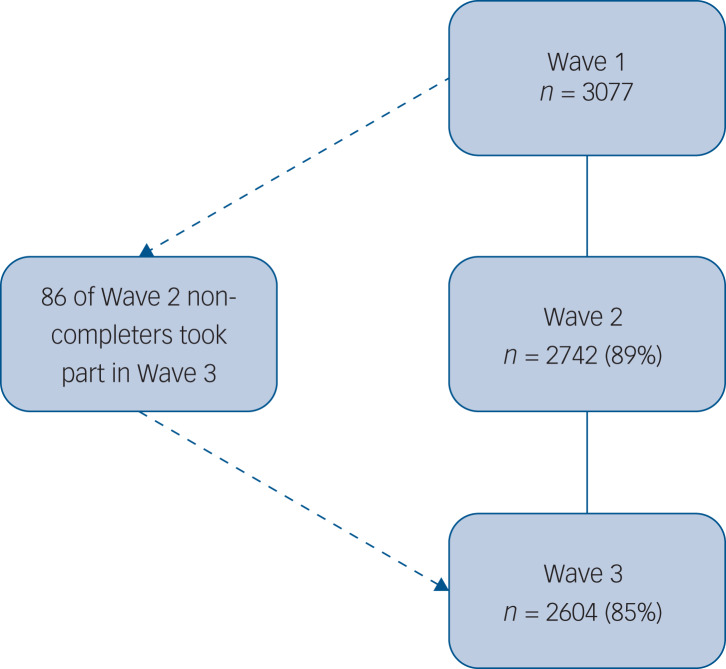
Flow of participants across waves.

Three additional waves were scheduled between end of May and autumn 2020, with longer-term follow-ups also anticipated. This data collection schedule was decided to minimise fatigue effects and to maximise follow-up over time. In addition, we anticipated that after the initial shock of lockdown, changes in participants’ well-being may be less marked over time thereby not requiring weekly data collection. Findings for waves 1 (31 March to 9 April 2020), 2 (10 April to 27 April 2020) and 3 (28 April to 11 May 2020) are reported here. At wave 2, 89% (*n* = 2742) completed the survey and at wave 3 the survey was completed by 85% (*n* = 2604; see Fig. 1 for flow chart of participants across the waves).

The authors assert that all procedures contributing to this work comply with the ethical standards of the relevant national and institutional committees on human experimentation and with the Helsinki Declaration of 1975, as revised in 2008. All procedures involving human patients were approved by the University of Glasgow's Medical, Veterinary & Life Sciences Ethics Committee (approval number: 200190146). The study was pre-registered at aspredicted.org (#41910). Participants received £1.50 for the completion of the surveys and were entered into prize draws. A list of mental health support organisations was made available to participants online.

### Measures

Suicidal history was assessed via two items adapted from the Adult Psychiatric Morbidity Survey,^20^ ‘Have you ever made an attempt to take your life, e.g. by taking an overdose of tablets or in some other way?’ (yes/no) (suicide attempt history) and ‘Have you ever deliberately harmed yourself in any way but not with the intention of killing yourself?’ (yes/no) (self-harm history). If respondents answered yes to the suicide attempt or the self-harm history questions, they were asked ‘when was the last time you deliberately harmed yourself?’ (past week, past month, past 6 months, more than 6 months, more than 12 months). We report self-harm and suicide attempts in the past week. Suicidal ideation in the past week was assessed by the question ‘How often have you thought about taking your life in the last week? (‘one day’, ‘several days’, ‘more than half the days’, ‘nearly everyday’, ‘never’, ‘I would rather not answer’)’. ‘One day’ to ‘nearly everyday’ was coded as yes, ‘never’ as no.

Depressive symptoms were assessed via the nine-item Patient Health Questionnaire (PHQ-9^21^). The 7-item Generalized Anxiety Disorder (GAD-7)^22^ tool was used to assess symptoms of generalised anxiety disorder. Both measures ask how often symptoms are bothering the respondents in the past 2 weeks. Scores of ≥10 on both measures are thought to indicate moderate levels of depression and anxiety and are used as cut-off points here.^22,23^

Feelings of defeat (perceived failed struggle and loss of rank) were assessed using four items from Griffiths’ short-form scale.^24^ The Entrapment Scale Short-form^25^ was used to explore perceptions of entrapment (feeling trapped by thoughts and feelings or situation). Mental well-being was assessed via the 7-item Short Warwick Edinburgh Mental Well-Being Scale (SWEMWBS).^26^ Loneliness was assessed using the UCLA 3-item scale.^27^ The National Readership Survey social grade^18^ was used as an indicator of socioeconomic group (SEG): high (A + B + C1) versus low (C2 + D + E).

To assess pre-existing mental health conditions, participants were first asked if they had any long-standing physical or mental impairment, illness or disability. Participants were then asked to select their mental or physical impairment from a list of options, which included mental health conditions, neuro-divergent disorders and alcohol and drug problems, and these responses were used to create a dichotomous variable for presence or absence of a pre-existing mental health condition (see Table 2 of the Methods in the supplementary materials).

### Statistical analysis

All analyses were conducted using Stata MP 16. Our analyses were conducted using an imputed data-set of the 3077 participants who completed the survey at wave 1 as there were significant differences in the mental health of those who did and did not complete all waves of the survey. We used multiple imputation (MI) to generate 50 data-sets for each outcome variable. MI generalised estimating models (MI-GEE) were then constructed to test the changes in the variables across waves for the whole sample. This approach is suitable for longitudinal data.^28,29^ As a sensitivity check, we ran all analyses with and without MI and found a similar pattern for both analyses. The GEE results presented here are those using MI. GEE models use a multilevel approach and produce odds ratios (ORs).

For the binary outcome variables (suicidal ideation, PHQ-9 and GAD-7 cut-off scores) binomial logit modelling was used, and for the continuous outcome variables (defeat, entrapment, loneliness, and positive well-being) linear Gaussian identity modelling was used. Region (South England, English Midlands, North England, Scotland, Wales and Northern Ireland) was controlled for in all analyses. We modelled the temporal covariation using an unstructured correlation matrix, as the pattern of associations was neither fully exchangeable nor had an first-order autoregressive structure. Further GEE models were conducted to test for subgroup differences in the outcome variables: as a function of age (18–29, 30–59, ≥60 years), gender (men, women), ethnicity (categorised into White and minority ethnic group because of small numbers), SEG (higher, lower) and the presence of a pre-existing mental health problem (no, yes).

Additionally, interactions between each of these subgroups and changes in each outcome over the waves was also tested, with only significant interactions reported in the results. As before, the binary variables were analysed using binomial logit GEE models and the continuous variables were explored using linear Gaussian identity GEE models.

Given the large number of analyses, a false discovery rate (FDR) was applied to all the between, within and interaction *P*-values from all analyses. FDR is a method of understanding the rate of type I errors in null hypothesis testing when conducting multiple comparisons. FDR-controlling procedures are designed to control the expected proportion of ‘discoveries’ that are false.^30^ There were few missing data at wave 1; a small number of participants indicated that they ‘would rather not say’ for the suicidal ideation (*n* = 93, 3% at wave 1; *n* = 91, 3.3% at wave 2; *n* = 71, 2.7% at wave 3), suicide attempts (*n* = 71, 2.3% at wave 1; *n* = 36, 1.3% at wave 2; *n* = 32; 1.2% at wave 3) or self-harm (*n* = 64, 2.1% at wave 1; *n* = 39, 1.4% at wave 2; *n* = 33, 1.3% at wave 3) questions; these were imputed via MI. As the rates of self-harm and suicide attempts in the past week were found to be low, no inferential statistics were applied to these data.

## Results

### Participant characteristics

A total of 3077 adults completed the survey at wave 1 (see Table 1, Fig. 1). In the unweighted data at wave 1, 55.1% of the participants were women and 90.5% were White. Regarding age, 27.5% were aged 18–29 years, 53.2% aged 30–59 and 19.3% aged ≥60 years. In total, 59.6% were married/living with partner and 92.0% self-identified as heterosexual. Over half (57.1%) reported occupations that were classified as higher SEG and 59.6% reported owning their own home (including with mortgage). Just over a quarter (27.2%) of the participants reported having a pre-existing mental health problem at wave 1 (see Table S6 in the supplementary materials).

### Mental health outcomes across waves for all adults

Rates of suicidal ideation increased over time (see Table 2), with respondents at wave 2 (9.2%; OR = 1.17, 95% CI 1.01–1.34, *P* = 0.031) and wave 3 (9.8%; OR = 1.24, 95% CI 1.07–1.44, *P* = 0.005) reporting higher levels than at wave 1 (8.2%). The difference between waves 2 and 3 was not statistically significant.

**Table 2:** Changes in primary outcome variables over waves 1–3 with odds ratios (ORs) and 95% confidence interval

	Wave 1 (*n* = 3077)	Wave 2 (*n* = 2742)	Wave 3 (*n* = 2604)	OR (95% CI), *P*		
				Wave 1–wave 2^a^	Wave 1–wave 3^a^	Wave 2–wave 3^b^
Suicidal ideation past week, % (95% CI)	8.2 (7.2 to 9.2)	9.2 (8.1 to 10.3)	9.8 (8.7 to 10.9)	1.17 (1.01 to 1.34), 0.03	1.24 (1.07 to 1.44), 0.005	0.94 (0.81 to 1.09), 0.42
Suicide attempt past week, % (95% CI)	0.1 (–0.3 to 0.5)	0.8 (0.4 to 1.2)	0.7 (0.3 to 1.1)	N/A	N/A	N/A
Self- harm past week, % (95% CI)	0.7 (0.4 to 1.1)	1.8 (1.3 to 2.3)	1.4 (1.0 to 1.9)	N/A	N/A	N/A
PHQ-9, ≥10: % (95% CI)	26.1 (24.6 to 27.7)	24.3 (22.7 to 25.9)	23.7 (22.1 to 25.3)	0.94 (0.87 to 1.02), 0.13	0.93 (0.86 to 1.02), 0.12	1.01 (0.93 to 1.10), 0.84
GAD-7, % ≥10: % (95% CI)	21.0 (19.6 to 22.4)	18.6 (17.1 to 20.1)	16.8 (15.4 to 18.2)	0.89 (0.81 to 0.97), 0.012	0.82 (0.74 to 0.90), < 0.0001	1.09 (0.78 to 1.21), 0.125
Defeat, mean (95% CI)	4.11 (4.11 to 4.39)	4.02 (3.87 to 4.17)	3.92 (3.77 to 4.07)	0.84 (0.75 to 0.94), 0.003	0.80 (0.71 to 0.91), < 0.0001	1.15 (0.92 to 1.19), 0.466
Entrapment, mean (95% CI)	3.96 (3.81 to 4.11)	3.78 (3.62 to 3.94)	3.60 (3.44 to 3.76)	0.88 (0.78 to 1.00), 0.04	0.79 (0.69 to 0.91), 0.001	1.11 (0.97 to 1.28), 0.14
Loneliness, mean (95% CI)	5.24 (5.17 to 5.31)	5.18 (5.11 to 5.25)	5.15 (5.08 to 5.22)	0.97 (0.92 to 1.03), 0.304	0.96 (0.90 to 1.02), 0.18	1.01 (0.95 to 1.08), 0.705
Well-being, mean (95% CI)	22.27 (22.05 to 22.49)	22.64 (22.41 to 22.87)	22.92 (22.68 to 23.16)	1.30 (1.09 to 1.58), 0.005	1.58 (1.29 to 1.92), <0.0001	0.83 (0.68 to 1.2), 0.078

N/A not applicable; PHQ-9, nine-item Patient Health Questionnaire; GAD-7, seven-item Generalized Anxiety Disorder.

a. Reference group: wave 1.

b. Reference group: wave 3

The rates of suicide attempt (0.1% at wave 1, 0.7% at wave 3) and self-harm (0.7% at wave 1 and 1.4% at wave 3) in the past week were low.

A total of 21% of the participants was above the cut-off point for moderate or severe levels of symptoms of anxiety at wave 1. However, these symptoms decreased across waves, with wave 2 (18.6%; OR = 0.89, 95% CI 0.81–0.97, *P* = 0.012) and wave 3 (16.8%; OR = 0.82, 95% CI 0.74–0.90, *P* < 0.0001) being lower than wave 1 (21%); the differences between wave 2 and wave 3 was not significant. More than a quarter (26.1%) scored above the cut-off for moderate or severe levels of depression; there was no significant change across the waves.

Feelings of defeat decreased from wave 1 (mean 4.11) to wave 2 (mean  4.02; OR = 0.84, 95% CI 0.75–0.95, *P* = 0.003) and from wave 1 to wave 3 (mean 3.92; OR = 0.80, 95% CI 0.71–0.91, *P* < 0.0001; Table 2). There was no difference between waves 2 and 3. Entrapment also decreased over time, from wave 1 (mean 3.96) to wave 2 (mean 3.78; OR = 0.88, 95% CI 0.79–1.00, *P* = 0.04) and from wave 1 to wave 3 (mean 3.60; OR = 0.79, 95% CI 0.69–0.91, *P* = 0.001) but not between waves 2 and 3. Positive well-being increased from wave 1 (mean 22.27) to wave 2 (mean 22.64; OR = 1.30, 95% CI 1.09–1.58, *P* = 0.005) and from wave 1 to wave 3 (mean 22.92; OR = 1.58, 95% CI 1.29–1.92, *P* < 0.0001), but not from wave 2 to wave 3. Levels of loneliness did not significantly change over waves.

### Mental health outcomes across wave by sociodemographic characteristics and pre-existing mental health condition

Percentages and means from wave 1 have been used to illustrate differences between subgroups, although data from all waves were used in this analysis and are included in the supplementary material (see supplementary Tables 3–8).

#### Suicidal ideation

Men and women reported similar levels of suicidal ideation (see supplementary Table 3). Compared with younger adults (18–29 year olds; wave 1 12.5%) those aged 30–59 years (8.4%; OR = 0.65, 95% CI 0.49–0.85, *P* = 0.002) and ≥60 years (1.9%; OR = 0.14, 95% CI 0.08–0.27, *P* < 0.0001) reported lower levels of suicidal ideation, and those aged 30–59 were more likely to report suicidal ideation than ≥60-year-olds (OR = 4.51, 95% CI 2.43–8.39, *P* < 0.0001). There were no clear differences when comparing ethnic minorities to the White ethnic group (see supplementary Table 4). Those from the lower SEG were more likely to experience suicidal ideation (10.3%; OR = 1.63, 95% CI 1.24–2.10, *P* < 0.0001) compared with those in the higher SEG (6.6%; see supplementary Table 5). Those with a pre-existing mental health condition were more likely to experience suicidal ideation (19.3%; OR = 5.56, 95% CI 4.23–7.31, *P* < 0.0001) compared with those without (4.1%; see supplementary Table 6).

#### Depressive symptoms

Men reported lower levels of depressive symptoms (17.6%) than women (33%; OR = 0.44, 95% CI 0.37–0.52, *P* < 0.0001; see supplementary Table 3). Those who were aged 30–59 years (26%; OR = 0.55, 95% CI 0.46–0.66, *P* < 0.0001) and aged ≥60 years (8.2%; OR = 0.14, 95% CI 0.10–0.20, *P* < 0.0001) reported lower levels of depressive symptoms than younger adults (38.8%; 18–29 years), and those aged 30–59 years reported higher rates of depressive symptoms than those ≥60 (OR = 3.85, 95% CI 2.82–5.27, *P* < 0.0001). There were no significant differences by ethnicity. The respondents in the lower SEG were more likely to report higher levels of depressive symptoms (30.4%; OR = 1.47, 95% CI 1.25–1.73, *P* < 0.0001) than those in the higher SEG group (22.9%; see supplementary Table 5).

People with a pre-existing mental health condition were significantly more likely to report higher levels of depressive symptoms (54.2%; OR = 6.50, 95% CI 5.45–7.77, *P* < 0.0001) compared with those without (15.3%). The interaction between pre-existing mental health condition and wave was statistically significant (OR = 0.87, 95% CI 0.79–0.96, *P* = 0.007), with those who had a pre-existing mental health condition reporting reductions in depressive symptoms over time at both wave 2 (reduction  5.6%; OR = 0.80, 95% CI 0.67–0.96, *P* = 0.017) and wave 3 (reduction 7.5%; OR = 0.76, 95% CI 0.63–0.94, *P* = 0.009), but not from wave 2 to wave 3 (reduction 1.9%). Those with no pre-existing mental health conditions did not change over time.

#### Anxiety symptoms

Across the three waves, those aged 30–59 years (21.5%; OR = 0.63, 95% CI 0.52–0.76, *P* < 0.0001) and those aged ≥60 (6.4%; OR = 0.16, 95% CI 0.11–0.23, *P* < 0.0001) were less likely to score above the cut-off for anxiety symptoms compared with those aged 18–29 years (30.1%), and those age 30–59 years were more likely to be above the cut-off for anxiety symptoms than those ≥60 (OR = 3.95, 95% CI 2.79–5.61, *P* < 0.0001; supplementary Table 3). Men were also less likely to meet the cut-off threshold (13%) compared with women (27.5%; OR = 0.40, 95% CI 0.33–0.48, *P* < 0.0001). Levels of anxiety did not vary by ethnicity. Those who were of a lower SEG were more likely to score above the cut-off for anxiety symptoms (24.9%; OR = 1.49, 95% CI 1.25–1.78, *P* < 0.0001) compared with those in the higher SEG (18%). Participants with a pre-existing mental health condition were more likely to score above the cut-off (44.6%; OR = 5.97, 95% CI 4.95–7.19, *P* < 0.0001) than those with no mental health condition (11.9%; supplementary Table 6).

#### Defeat

Compared with those aged 18–29 years (mean 5.27), participants aged 30–59 years (mean 4.38; OR = 0.40, 95% CI 0.29–0.55, *P* < 0.0001) and those aged ≥60 years (mean 2.45; OR = 0.06, 95% CI 0.04–0.09, *P* < 0.0001) reported lower levels of defeat, and 30–59 year olds scored higher than those aged ≥60 years (OR = 6.77, 95% CI 4.72–9.71, *P* < 0.0001; supplementary Table 7). Men reported significantly lower levels of defeat than females (OR = 0.22, 95% CI 0.17–0.29, *P* < 0.0001). No differences were found by ethnicity on levels of defeat. Participants from a lower SEG reported higher levels of defeat (mean 4.81; OR = 2.69, 95% CI 2.03–3.56, *P* < 0.0001) compared with those of a higher SEG (mean 3.83; see supplementary Table 8). Participants who reported a pre-existing mental health condition reported higher levels of defeat (mean 7.06; OR = 48.47, 95% CI 36.56–64.27, *P* < 0.0001) compared with those without a mental health condition (mean 3.17; see supplementary Table 8).

#### Entrapment

Men reported lower levels of entrapment (mean 3.14) than women (mean 4.62; OR = 0.23, 95% CI 0.17–0.31, *P* < 0.0001). Levels of entrapment differed significantly by age group, with those aged 30–59 years (mean 4.1; OR = 0.37, 95% CI 0.26–0.53, *P* < 0.0001) and those aged ≥60 years (mean 1.93; OR = 0.05, 95% CI 0.03–0.07, *P* < 0.0001) reporting lower levels of entrapment than those aged 18–29 years (mean 5.07), and those aged 30–59 years were higher than those aged ≥60 (OR = 8.30, 95% CI 5.58–12.34, *P* < 0.0001). No significant differences by ethnicity were found. Those from a lower SEG reported significantly higher levels of entrapment (mean 4.47; OR = 2.48, 95% CI 1.82–3.38, *P* < 0.0001) than those in the higher SEG group (mean 3.57). Participants with a pre-existing mental health condition reported higher levels of entrapment (mean 7.0; OR = 66.78, 95% CI 48.99–91.04, *P* < 0.0001) than those without (mean 2.79). The interaction between mental health condition and entrapment over the waves was significant (OR = 0.84, 95% CI 0.72–0.98, *P* = 0.02), with those who had a mental health condition experiencing a more pronounced reduction in the average entrapment score from wave 1 to wave 3 (reduction 0.63; OR = 0.70, 95% CI 0.52–0.95, *P* = 0.02) compared with those with no mental health condition (reduction 0.2).

#### Loneliness

Men reported significantly lower levels of loneliness (mean 4.89) than women (mean 5.52; OR = 0.54, 95% CI 0.47–0.62, *P* < 0.0001). There were significant differences between the age groups on levels of loneliness, with those aged 30–59 years (mean 5.28; OR = 0.54, 95% CI 0.46–0.63, *P* < 0.0001) and those aged ≥60 (mean 4.31; OR = 0.21, 95% CI 0.17–0.26, *P* < 0.0001) reporting lower levels of loneliness than those aged 18–29 years (mean 5.87), and those aged 30–59 years reporting higher loneliness than the ≥60 group (OR = 2.54, 95% CI 2.13–3.02, *P* < 0.0001). Additionally, the interaction between age and loneliness over the waves was significant (OR = 1.06, 95% CI 1.01–1.11, *P* = 0.016), as levels of loneliness reduced significantly for those aged 18–29 years from wave 1 to wave 2 (reduction 0.17; OR = 0.78, 95% CI 0.65–0.92, *P* = 0.004) and wave 1 to wave 3 (reduction 0.21; OR = 0.81, 95% CI 0.67–0.97, *P* = 0.004) compared with those aged ≥60 years, whose self-reported loneliness increased (wave 1 to wave 2 increase of 0.11).

Levels of loneliness did not differ by ethnic group. Those from the lower SEG (mean 5.39; OR = 1.31, 95% CI 1.14–1.50, *P* < 0.0001) reported significantly higher levels of loneliness compared with those from the higher SEG (mean 5.12). Participants with a pre-existing mental health condition reported significantly higher levels of loneliness than (mean 6.28; OR = 4.19, 95% CI 3.63–4.85, *P* < 0.0001) those without (mean 4.24; see supplementary Table 8). There was evidence of a wave × mental health problem interaction (OR = 0.89, 95% CI 0.83–0.96, *P* = 0.002), with a significant decrease in loneliness in those who had a pre-existing mental health problem from wave 1 to wave 3 (reduction 0.26; OR = 0.80, 95% CI 0.70–0.92, *P* = 0.002), but no significant changes for those with no mental health condition.

#### Positive well-being

Levels of well-being differed by age groups, with those aged 30–59 years (mean 22.01; OR = 5.78, 95% CI 3.52–9.49, *P* < 0.0001) and those aged ≥60 (mean 26.01; OR = 255.59, 95% CI 136.39–479.44, *P* < 0.0001) reporting higher levels of well-being than the 18- to 29-year-olds (mean  20.28). Men reported significantly higher well-being (mean 23.29) compared with women (mean 21.45; OR = 6.17, 95% CI 3.97–9.57, *P* < 0.0001). Levels of well-being were not significantly different by ethnic group. Those of a lower SEG reported lower levels of well-being (mean 21.75; OR = 0.41, 95% CI 0.26–0.64, *P* < 0.0001) compared with those of a higher SEG (mean  22.66). Participants with a pre-existing mental health condition were more likely to report lower well-being scores (mean 18.64; OR = 0.007, 95% CI 0.004–0.01, *P* < 0.0001) compared with those with none (mean 23.66; see supplementary Table 8).

## Discussion

### Main findings and comparison with findings from other studies

This study offers a detailed examination of the mental health and well-being of the UK adult population during the first 6 weeks of the COVID-19 pandemic. Across every indicator, individuals from more socially disadvantaged backgrounds and those with pre-existing mental health problems report the worst mental health outcomes. The rates of suicidal ideation increased during the initial weeks of lockdown, with one in seven (14%) young adults reporting suicidal thoughts in the last week at wave 3. It is not possible to make direct comparisons with pre-COVID-19 rates, but the rate of suicidal ideation among young adults reported here (between 12.5% and 14.4% across waves) is higher than the 11% past-year suicidal ideation rate reported by young adults in another pre-COVID-19 study.^31^ The weekly suicidal ideation rates for the whole sample (9.8% at wave 3) are also higher than those reported elsewhere, with 2.8% reporting suicidal thoughts in one national study of adults.^32^

Across all three waves, approximately one in four respondents (wave 1 = 26.1%, wave 2 = 24.3% and wave 3 = 23.7%) experienced moderate to severe levels of depressive symptoms on the PHQ-9. This finding is concerning when compared with other pre-COVID-19 general population studies where, for example, 5.6% scored above the standard ≥10 cut-off.^33^ However, we urge caution when extrapolating from the PHQ-9 data to general population lockdown estimates, as a recent meta-analysis concluded that the PHQ-9 may more than double the estimate of depression compared with a structured clinical interview (using the Structured Clinical Interview for Diagnostic and Statistical Manual of Mental Disorders).^34^ Also, although we have recruited a well-stratified national sample from across the UK, quota sampling does not guarantee the same level of representativeness as probability sampling and therefore prevalence estimates need to be interpreted accordingly. However, it is also worth noting that our depressive symptoms findings are quite similar to the latest Office for National Statistics data for the UK adult population collected in June 2020,^35^ where 19.2% of adults reported moderate to severe levels of depression, compared with 23.7% at wave 3 in our study at the end of April/start of May 2020.

For anxiety, one in five (21%) respondents in the present sample scored above the cut-off on the GAD-7, corresponding to moderate to severe levels of anxiety at wave 1, with this rate decreasing to 16.8% by wave 3. We do not have pre-COVID-19 figures to make like-for-like comparisons; nonetheless, these rates were much higher than the established general population norms (of approximately 5%).^36^ Levels of mental well-being among women across all waves were lower than the general population norms for the SWEMWBS, but levels for men were similar.^26^

### Implications

As already noted, the mental health of women, of young people (18–29 years), of those from more socially disadvantaged backgrounds, and of those with pre-existing mental health problems has been particularly affected during the pandemic. These groups need to be prioritised to ensure that they receive the support they require^16^ and accessible and remote clinical services tailored, as necessary, to meet this need. The trajectories across the three waves illustrate the importance of assessing different indicators of mental health and well-being. Whereas symptoms of anxiety, and levels of defeat and entrapment decreased across the three waves, depressive symptoms and loneliness remained stable but adversely affected. The findings also highlight that loneliness may become more of an issue for older adults as the pandemic unfolds, as well as for those from more socially disadvantaged backgrounds.

Across all of the analyses the mental health outcomes for those from ethnic minority and White backgrounds were similar. Despite our sample being well-stratified nationally, our sample size precludes a more fine-grained analysis of the mental health outcomes of people from specific ethnic minority communities. Such an analysis, that also takes account of intersectionality, is urgently required.^1,16,17^

The trajectories of suicidal thoughts highlight that we need to be vigilant. Although an increase in suicide is not inevitable,^37^ the present data may be an early indicator of emerging risk, especially as the economic fallout of COVID-19 escalates. The proportions of respondents reporting at least 1 day in the previous week that they wanted to end their life increased across the three waves of the study, from 8.2%, to 9.2% and 9.8% at waves 1, 2 and 3, respectively.

Given its well established relationship with suicide risk,^38^ it is surprising that levels of entrapment decreased while suicidal thoughts increased. This may reflect a lagged effect or it may be that the items assessing entrapment or depression focus on the past whereas the suicidal question is tapping uncertainty or concerns about the future. This may also explain why the positive well-being measure increased, as it also focuses on the past, and likely increased as levels of anxiety decreased. The focus on future orientation is potentially crucial as future thinking is a recognised cognitive factor associated with suicidal ideation independent of depression.^38^ Indeed, inspection of the items to assess depressive symptoms illustrates this point as they are tapping the extent to which respondents are ‘bothered’ by problems in the recent past; so after the initial shock of lockdown, one's appraisal of these problems is relatively stable in the short term. By contrast, in the early weeks of the pandemic, the anticipated impact of the economic and social disruption to come may have exacerbated one's feelings of hopelessness and suicidal ideation and hence explain the increase in the latter.

Survey-based research needs to be supplemented with qualitative interviews to determine whether our conjecture about the cause of increasing levels of suicidal ideation is supported. By way of *post hoc* analyses, we also inspected the responses to the suicidal question in the PHQ-9 (‘Thoughts that you would be better off dead, or of hurting yourself in some way?’) and we find a similar pattern as above, of increasing suicidal thoughts. It is essential, therefore, that suicidal thoughts continue to be tracked as we emerge from lockdown and navigate national/local restrictions. These data are also consistent with the recent report from the National Child Mortality Database that points to a potential increase in child suicide deaths in the early stages of the pandemic.^39^ The defeat and entrapment levels are also of concern, especially among young adults at wave 1. At wave 1, more than a third (37%) of young people scored above the recommended cut-off (>5) for entrapment, which indicates that further screening for suicide ideation is warranted.^25^

### Limitations

Indicators of mental health were based on self-reports rather than clinical diagnoses, as a result, we can only comment on the trajectory of the symptoms of mental ill health rather than psychiatric disorder. Despite successfully recruiting a quota-based national sample, similar to all studies that recruit via digital means, our sample is likely to underestimate the mental health effects of COVID-19 as those who are digitally excluded may be underrepresented. Also, those who did not complete all waves tended to have worse mental health at wave 1. Future research is required to understand what aspects of the pandemic and the pandemic response may have contributed to negative mental health outcomes as well as those factors and activities that may be protective.

To conclude, the mental health and well-being of the UK adult population appears to have been affected in the initial phase of the COVID-19 pandemic, particularly women, young adults, the socially disadvantaged and those with pre-existing mental health problems. The trajectory of increasing rates of suicidal thoughts, especially among young adults, is particularly concerning. These early data highlight that detailed monitoring of longer-term mental health outcomes and inequalities is essential.

## Data Availability

The data that support the findings of this study are available from the corresponding author (R.C.O'C.), upon reasonable request.

## Supplementary material

For supplementary material accompanying this paper visit http://dx.doi.org/10.1192/bjp.2020.212. click here to view supplementary material
