# Supplementary material for: Mental health and well-being during the COVID-19 pandemic: longitudinal analyses of adults in the UK COVID-19 Mental Health & Wellbeing study
Source: Br J Psychiatry. 2020 Oct 21:1–8. doi: 10.1192/bjp.2020.212 (PMC7684009; doi:10.1192/bjp.2020.212)
Supplement: Supplementary file 1 [file S0007125020002123sup001.docx]

**Supplementary materials**

**List of Tables**

Table S1: Breakdown of gender identity of participants (n=3077)

Table S2: Breakdown of participants by region of UK (n=3077)

Table S3: Recent suicidal history, depressive symptoms and anxiety symptoms by age group and gender Table S4: Recent suicidal history and depressive symptoms and anxiety symptoms cut-off scores by ethnicity (White vs BAME) Table S5: Recent suicidal history, depressive symptoms and anxiety symptoms cut-off scores by socio-economic group (SEG) Table S6: Recent suicidal history and depressive symptoms and anxiety symptoms cut-off scores by presence any mental health (MH) problems Table S7: Means and 95% confidence intervals [CIs] of variables by grouping variable over waves (n=3077)

**Methods**

1. Full details of measures
2. Breakdown of pre-existing mental health problems
3. Differences between participants who completed all waves compared to those who did not on wave 1 measures

**Table S1: Breakdown of gender identity of participants (n=3077)**

| **Gender identity** | | | | | | | | |
| --- | --- | --- | --- | --- | --- | --- | --- | --- |
| Age group, years | | | | | | | | |
|  | 18-29, % [95% CI] | | 30-59, % [95% CI] | | 60+, % [95% CI] | | Total, % [95% CI] | |
|  | Not weighted | Weighted | Not weighted | Weighted | Not weighted | Weighted | Not weighted | Weighted |
| Female | 72 [67.0-75.0] | 68.4 [65.3-71.5] | 52 [49.6-54.4] | 47 [44.5-49.5] | 37.7 [33.8-41.6] | 34.2 [30.4-38.0] | 54.8 [53.0-56.6] | 50.7 [48.9-52.5] |
| Male | 27.4 [24.4-30.4] | 31 [27.9-34.1] | 47.9 [45.5-50.3] | 52.9 [50.4-55.4] | 62.3 [58.4-66.2] | 65.8 [62.0-69.6] | 45 [43.2-46.8] | 49.1 [47.3-50.9] |
| TG female | 1 [0.3-1.7] | 0.1 [-0.6-0.8] | 0.1 [-0.4-0.6] | 0.1 [-0.4-0.6] | 0 [0.0-0.0] | 0 [0.0-0.0] | 0.1 [-0.3-0.5] | 0.1 [-0.3-0.5] |
| TG male | 1 [0.3-1.7] | 0.1 [-0.6-0.8] | 0 [0.0-0.0] | 0 [0.0-0.0] | 0 [0.0-0.0] | 0 [0.0-0.0] | 0.1 [-0.3-0.5] | 0.1 [-0.3-0.5] |
| Gender variant | 3 [1.9-4.2] | 0.3 [-0.4-1.0] | 0 [0.0-0.0] | 0 [0.0-0.0] | 0 [0.0-0.0] | 0 [0.0-0.0] | 0.1 [-0.3-0.5] | 0.1 [-0.3-0.5] |

Note: TG=transgender

**Table S2: Breakdown of participants by region of UK (n=3077)**

|  | Age group, years | | | | | | | |
| --- | --- | --- | --- | --- | --- | --- | --- | --- |
|  | **18-29, % [95% CI]** | | **30-59, % [95% CI]** | | **60+, % [95% CI]** | | **Total, % [95% CI]** | |
|  | Not weighted | Weighted | Not weighted | Weighted | Not weighted | Weighted | Not weighted | Weighted |
| UK Region |  |  |  |  |  |  |  |  |
| South West | 7.9 [6.1-9.7] | 8.5 [6.7-10.4] | 7.1 [5.9-8.3] | 7.9 [6.6-9.3] | 10.6 [8.1-13.1] | 12 [9.4-14.6] | 8 [7.0-9.0] | 8.9 [7.9-9.9] |
| South East | 17.9 [15.3-20.5] | 15.4 [13.0-17.8] | 16 [14.2-17.8] | 13.9 [12.2-15.6] | 16.3 [13.3-19.3] | 14.6 [11.8-17.4] | 16.6 [15.3-17.9] | 14.5 [13.2-15.8] |
| East Anglia | 6.5 [4.8-8.2] | 4 [2.7-5.3] | 6.9 [5.7-8.1] | 4.4 [3.4-5.4] | 5.6 [3.8-7.5] | 3.5 [2.0-5.0] | 6.5 [5.6-7.4] | 4.1 [3.4-4.8] |
| West Midlands | 8.4 [6.5-10.3] | 10.9 [8.8-13.0] | 5.7 [4.6-6.8] | 7.3 [6-8.6] | 8.6 [6.4-10.9] | 11.6 [9.0-14.2] | 7 [6.1-7.9] | 9.2 [8.2-10.2] |
| East Midlands | 6.6 [4.9-8.3] | 8.5 [6.7-10.4] | 5.3 [4.2-6.4] | 6.8 [5.5-8.1] | 7.2 [5.1-9.3] | 10 [7.6-12.4] | 6 [5.2-6.8] | 7.9 [6.9-8.9] |
| Yorkshire /Humbershire | 10.4 [8.3-12.5] | 9.2 [7.3-11.1] | 9.5 [8.1-10.9] | 8.5 [7.1-9.9] | 8.9 [6.6-11.2] | 8 [5.8-10.2] | 9.6 [8.6-10.6] | 8.6 [7.6-9.6] |
| North West | 12.8 [10.6-15.1] | 11.1 [9.0-13.2] | 13.8 [12.1-15.5] | 12.5 [10.8-14.2] | 11.1 [8.6-13.6] | 10.5 [8.1-13.0] | 13 [11.8-14.2] | 11.7 [10.6-12.9] |
| North East | 5.7 [4.1-7.3] | 5.1 [3.6-6.6] | 4.6 [3.6-5.6] | 3.8 [2.8-4.8] | 4.5 [2.8-6.2] | 4 [2.4-5.6] | 4.9 [4.1-5.7] | 4.2 [3.5-4.9] |
| Scotland | 6.6 [4.9-8.3] | 5.1 [3.6-6.6] | 11.6 [10.1-13.2] | 8.8 [7.4-10.2] | 17.7 [14.6-20.8] | 13.6 [10.9-16.3] | 11.4 [10.3-12.5] | 8.7 [7.7-9.7] |
| Wales | 4.8 [3.4-6.2] | 5.4 [3.9-6.9] | 4.4 [3.4-5.4] | 5 [3.9-6.1] | 3.9 [2.3-5.5] | 4.5 [2.8-6.2] | 4.4 [3.7-5.1] | 5 [4.2-5.8] |
| Northern Ireland | 1.4 [0.6-2.2] | 2.2 [1.2-3.2] | 2.9 [2.1-3.7] | 4.4 [3.4-5.443] | 0.3 [-0.5-1.1] | 0.7 [-0.1-1.5] | 2 [1.5-2.5] | 3 [2.4-3.6] |
| Greater London | 11 [8.9-13.1] | 14.6 [12.3-17.0] | 12.4 [10.8-14.0] | 16.8 [14.943-18.7] | 5.2 [3.4-7.0] | 7 [5.0-9.0] | 10.6 [9.5-11.7] | 14.2 [13.0-15.5] |

**Table S3. Recent suicidal history, depressive symptoms and anxiety symptoms by age group and gender**

|  | Wave 1 (n=3077) | | | | Wave 2 (n=2742) | | | | | Wave 3 (n=2604) | | | | |
| --- | --- | --- | --- | --- | --- | --- | --- | --- | --- | --- | --- | --- | --- | --- |
|  | 18- 29 yrs | 30- 59 yrs | 60+ yrs | Total | 18- 29 yrs | 30- 59 yrs | 60+ yrs | Total | 18- 29 yrs | | 30- 59 yrs | 60+ yrs | Total |  |
|  | % [95% CI] | % [95% CI] | % [95% CI] | % [95% CI] | % [95% CI] | % [95% CI] | % [95% CI] | % [95% CI] | % [95% CI] | | % [95% CI] | % [95% CI] | % [95% CI] |  |
| Men |  |  |  |  |  |  |  |  |  | |  |  |  |  |
| Suicidal ideation last week | 12.3  [11.1 – 13.5] | 8.8  [7.8- 9.8] | 2.5 [1.9- 3.1] | 7.7 [6.8- 8.6] | 14.6 [13.3- 15.9] | 9.5 [8.4- 10.6] | 4.1 [3.4- 4.8] | 8.7 [7.7- 9.8] | 14.1 [12.8- 15.4] | | 11.4 [10.2- 12.6] | 3.2 [2.5- 3.9] | 9.4 [8.3- 10.5] |  |
| Suicide attempt last week | 0 [0- 0] | 0.1 [-0.3 – 0.5] | 0 [0.0- 0.0] | 0.1 [-0.3 – 0.5] | 0 [0.0- 0.0] | 0.8 [0.4- 1.2] | 0 [0.0- 0.0] | 0.5 [0.1- 0.9] | 0 [0.0- 0.0] | | 1.0 [0.6- 1.4] | 0 [0.0- 0.0] | 0.6 [0.2- 1.0] |  |
| Self- harm last week | 0.5 [0.2- 0.9] | 0.4 [0.1- 0.8] | 0 [0.0- 0.0] | 0.3 [-0.1 – 0.7] | 2.9 [2.3- 3.5] | 1.5 [1.1 – 2.0] | 0.3 [-0.1 – 0.7] | 1.4 [1.0- 1.8] | 1.8 [1.3- 2.3] | | 1.3 [0.9- 1.7] | 0 [0.0- 0.0] | 1.0 [0.6- 1.4] |  |
| PHQ9 (% ≥ 10)^a^ | 25.2[19.6-30.8] | 20.3[17.1-23.5] | 7[4.4-9.6] | 17.6[15.6-19.6] | 25[18.6-31.4] | 20.2[17.3-23.1] | 9.5[6.4-12.6] | 17.9[15.8-20.0] | 22.8[16.4-29.2] | | 20.5[17.5-23.5] | 6.9[4.2-9.6] | 16.9[14.8-19.0] |  |
| GAD7 (% ≥ 10)^b^ | 18.3[13.3-23.3] | 15.3[12.5-18.1] | 4.6[2.5-6.7] | 13[11.2-14.8] | 17[11.5-22.6] | 13.8[11.3-16.3] | 6.3[3.8-8.9] | 12.2[10.4-14.0] | 14.4[9.1-19.7] | | 12.7[10.2-15.2] | 4.6[2.4-6.8] | 10.6[8.9-12.3] |  |
| Women |  |  |  |  |  |  |  |  |  | |  |  |  |  |
| Suicidal ideation last week | 12.5 [11.3- 13.7] | 7.9 [7.0 – 8.9] | 0.9 [0.6- 1.3] | 8.6 [7.6- 9.6] | 14.2 [12.9- 15.5] | 9.4 [8.3- 10.5] | 0 [0.0- 0.0] | 9.6 [8.5- 10.7] | 14.5 [13.2- 15.9] | | 9.9 [8.8- 11.1] | 1.9 [1.4- 2.4] | 10.1 [8.9- 11.3] |  |
| Suicide attempt last week | 0.2 [-0.2- 0.6] | 0.2 [-0.2- 0.6] | 0 [0.0- 0.0] | 0.2 [-0.2- 0.6] | 2.0 [1.5- 2.5] | 0.8 [0.4- 1.2] | 0 [0.0- 0.0] | 1.1 [0.7- 1.5] | 1.4 [1.0.- 1.9] | | 0.6 [0.2- 1.0] | 0 [0.0- 0.0] | 0.7 [0.3- 1.1] |  |
| Self- harm last week | 1.3 [0.9- 1.7] | 1.2 [0.8 – 1.6] | 0 [0.0- 0.0] | 1.1 [0.7- 1.5] | 3.8 [3.1- 4.5] | 1.5 [1.1 – 2.0] | 0.9 [0.5- 1.3] | 2.2 [1.7- 2.8] | 3.0 [2.3- 3.7] | | 1.2 [0.8- 1.6] | 0.5 [0.1- 0.9] | 1.7 [1.2- 2.2] |  |
| PHQ-9 (% ≥ 10) | 43.8[39.9-47.7] | 31.3[28.2-34.4] | 10.2[6.3-14.2] | 33[30.8-35.2] | 39.8[35.6-44.0] | 27.6[24.4-30.8] | 12.7[8.2-17.2] | 29.7[27.4-32.0] | 38.2[33.7-42.7] | | 29.9[26.5-33.3] | 10[5.9-14.1] | 29.6[27.2-32.0] |  |
| GAD-7 (% ≥ 10) | 34.6[30.8-38.4] | 27.1[24.1-30.1] | 9.3[5.5-13.1] | 27.5[25.4-29.6] | 28.8[24.9-32.7] | 24.1[21.1-27.1] | 10.8[6.6-15.0] | 23.9[21.7-26.1] | 29.3[25.1-33.5] | | 22.1[19.0-25.2] | 6.7[3.3-10.1] | 22.1[19.9-24.3] |  |
| All adults |  |  |  |  |  |  |  |  |  | |  |  |  |  |
| Suicidal ideation last week | 12.5 [11.3- 13.7] | 8.4 [7.4- 9.4] | 1.9 [1.4- 2.4] | 8.2 [7.2- 9.2] | 14.3 [13.0- 15.6] | 9.5 [8.4- 10.6] | 2.5 [1.9- 3.1] | 9.2 [8.1- 10.3] | 14.4 [13.1- 15.8] | | 10.6 [9.5- 11.9] | 2.7 [2.1- 3.3] | 9.8 [8.7- 10.9] |  |
| Suicide attempt last week | 0.1 [-0.3 – 0.5] | 0.2 [-0.2- 0.6] | 0.0 [0- 0] | 0.1 [-0.3 – 0.5] | 1.5 [1.1- 2.0] | 0.8 [0.4- 1.2] | 0 [0.0- 0.0] | 0.8 [0.4- 1.2] | 1.0 [0.6- 1.4] | | 0.8 [0.4- 1.2] | 0 [0.0- 0.0] | 0.7 [0.3- 1.1] |  |
| Self- harm last week | 1.1 [0.7- 1.5] | 0.8 [0.5- 1.2] | 0.0 [0- 0] | 0.7 [0.4- 1.1] | 3.6 [2.9- 4.3] | 1.5 [1.1 – 2.0] | 0.5 [0.1- 0.9] | 1.8 [1.3- 2.3] | 2.7 [2.1- 3.3] | | 1.3 [0.9- 1.7] | 0.2 [-0.2- 0.6] | 1.4 [1.0- 1.9] |  |
| PHQ-9 (% ≥ 10) | 38.8[35.5-42.1] | 26[23.9-28.1] | 8.2[6.0-10.4] | 26.1[24.6-27.7] | 36.1[32.5-39.7] | 24[21.8-26.2] | 10.7[8.1-13.3] | 24.3[22.7-25.9] | 34[30.3-37.7] | | 25.3[23.1-27.6] | 8.1[5.8-10.4] | 23.7[22.1-25.3] |  |
| GAD-7 (% ≥ 10) | 30.1[27.0-33.2] | 21.5[19.1-23.5] | 6.4[4.4-8.4] | 21[19.6-22.4] | 25.8[22.5-29.1] | 19.1[17.1-21.1] | 8.0[5.8-10.3] | 18.6[17.1-20.1] | 25.3[21.9-28.7] | | 17.5[15.5-19.5] | 5.4[3.5-7.3] | 16.8[15.4-18.2] |  |

**Table S4: Recent suicidal history and depressive symptoms and anxiety symptoms cut-off scores by ethnicity (White vs Black Asian and Minority Ethnic, BAME)**

| Variable | Wave 1 (n=3069) | | Wave 2 (2735) | | Wave 3 (2598) | |
| --- | --- | --- | --- | --- | --- | --- |
|  | **White (n=2777)** | **BAME (n=292)** | **White (n= 2499)** | **BAME (236)** | **White (2385)** | **BAME (213)** |
| Last week | % [95% CI] | % [95% CI] | % [95% CI] | % [95% CI] | % [95% CI] | % [95% CI] |
| Suicidal ideation | 8.1 [7.8- 8.4] | 9.5 [6.3-12.7] | 8.9 [8.6-9.2] | 13.8 [9.4-18.2] | 9.3 [9.0- 9.6] | 15.5 [10.8-20.2] |
| Suicide attempt | 0.1 [-0.01-0.2] | 0 [0.0- 0.0] | 0.8 [0.7-0.9] | 0.9 [-0.3-2.1] | 0.6 [0.5-0.7] | 1.4 [-0.1-2.9] |
| Self- harm | 0.8 [0.7-0.9] | 0 [0.0-0.0] | 1.9 [1.7-2.1] | 0.4 [-0.8-1.6] | 1.3 [1.2-1.4] | 1.9 [0.1-3.7] |
| Last 2 weeks |  |  |  |  |  |  |
| PHQ-9 (% ≥ 10) | 25.8 [25.3-26.3] | 29.1 [24.1- 34.1] | 24.1 [23.6-25.6] | 26.7 [21.3-32.1] | 23.6 [23.1-24.1] | 23.9 [18.4-29.4] |
| GAD-7 (% ≥ 10) | 20.8 [20.3-21.3] | 21.9 [17.4-26.4] | 18.7 [18.3-19.2] | 16.5 [12.0-21.0] | 16.8 [16.4-17.2] | 16.4 [11.6-21.2] |

PHQ-9 = Patient health questionnaire (depressive symptoms) cut-off score ≥ 10 indicates major depression, GAD-7 = Generalised anxiety disorder 7-item cut-off score ≥10 indicates moderate anxiety

**Table S5: Recent suicidal history, depressive symptoms and anxiety symptoms cut-off scores by socio-economic group (SEG)**

| Variable | Wave 1 (3077) | | | Wave 2 (2742) | | | Wave 3 (2604) | | |
| --- | --- | --- | --- | --- | --- | --- | --- | --- | --- |
|  | **High SEG (n=1758)** | **Low SEG (n=1319)** | **High SEG (1571)** | | **Low SEG (1171)** | **High SEG (1489)** | | **Low SEG (1115)** |  |
| Last week | % [95% CI] | % [95% CI] | % [95% CI] | | % [95% CI] | % [95% CI] | | % [95% CI] |  |
| Suicidal ideation | 6.6 [5.8-7.4] | 10.3 [9.0-11.6] | 8.3 [7.4- 9.2] | | 10.6 [8.0-12.0] | 8.9 [8.0-9.9] | | 11.0 [9.6-12.4] |  |
| Suicide attempt | 0.1 [-0.2-0.7] | 0.2 [-0.2-0.6] | 0.6 [0.3-.9] | | 1.0 [0.6- 1.4] | 0.8 [0.5-1.1] | | 0.5 [0.1- 1.0] |  |
| Self- harm | 0.4 [-0.1-0.7] | 1.3 [0.8-1.8] | 1.6 [1.2-2.0] | | 2.1 [1.5-2.7] | 1.5 [1.1-1.9] | | 1.2 [0.7-1.7] |  |
| Last 2 weeks |  |  |  | |  |  | |  |  |
| PHQ-9 (% ≥ 10) | 22.9 [21.6-24.2] | 30.4 [28.5-32.3] | 21.6 [20.3-22.9] | | 28.1 [26.2 -30.1] | 20.8 [19.5-22.2] | | 27.5 [25.5- 29.5] |  |
| GAD-7 (% ≥ 10) | 18.0 [16.8-19.2] | 24.9 [23.1-26.7] | 16.9 [15.7-18.1] | | 20.8 [19.0 –22.6] | 14.4 [13.2-15.6] | | 19.8 [18.0- 21.6] |  |

PHQ-9 = Patient health questionnaire (depressive symptoms) cut-off score ≥ 10 indicates major depression, GAD-7 = Generalised anxiety disorder 7-item cut-off score ≥10 indicates moderate anxiety

**Table S6: Recent suicidal history and depressive symptoms and anxiety symptoms cut-off scores by presence any mental health (MH) problems**

| Variable | Wave 1 (3077) | | Wave 2 (2742) | | Wave 3 (2604) | |
| --- | --- | --- | --- | --- | --- | --- |
|  | **No MH (n=2225)** | **MH (n= 852)** | **No MH (2002)** | **MH (740)** | **No MH (1906)** | **MH (698)** |
| Last week | % [95% CI] | % [95% CI] | % [95% CI] | % [95% CI] | % [95% CI] | % [95% CI] |
| Suicidal ideation | 4.1 [3.7-4.5] | 19.3 [17.0-21.6] | 5.8 [5.3-6.3] | 19.0 [16.5-21.5] | 6.0 [5.5-6.6] | 20.4 [17.8- 23.0] |
| Suicide attempt | 0.1 [-0.1- 0.3] | 0.2 [-0.4-0.8] | 0.7 [0.5-0.9] | 1.1 [0.5-1.8] | 0.6 [0.4-0.8] | 0.7 [0.1-1.3] |
| Self- harm | 0.3 [0.1-0.5] | 2.0 [1.2-2.8] | 1.0 [0.8-1.2] | 4.0 [2.7-5.2] | 0.9 [0.7-1.1] | 2.7 [1.7-3.8] |
| Last 2 weeks |  |  |  |  |  |  |
| PHQ-9 (% ≥ 10) | 15.3 [14.5-16.1] | 54.2 [51.4-57.1] | 15.4 [14.6-16.2] | 48.6 [45.5-51.7] | 15.3 [14.5-16.1] | 46.7 [43.5-49.9] |
| GAD-7 (% ≥ 10) | 11.9 [11.2-12.6] | 44.6 [41.8-47.4] | 11.2 [10.5-11.9] | 38.4 [35.4-41.4] | 9.9 [9.2-10.6] | 35.4 [32.4- 38.4] |

PHQ-9 = Patient health questionnaire (depressive symptoms) cut-off score ≥ 10 indicates major depression, GAD-7 = Generalised anxiety disorder 7-item cut-off score ≥10 indicates moderate anxiety

**Table S7. Means and 95% confidence intervals [CIs] of variables by age over waves (n=3077)**

| Variable | Wave 1 | | | Wave 2 | | | Wave 3 | | |
| --- | --- | --- | --- | --- | --- | --- | --- | --- | --- |
|  | **18-29 yrs (n=847)** | **30-59 yrs (n=1636)** | **60+ yrs (n=594)** | **18-29 yrs (n=694)** | **30-59 yrs (n=1488)** | **60+ yrs**  **(n=560)** | **18-29 yrs (n=618)** | **30-59 yrs (n=1431)** | **60+ yrs (n=555)** |
| Defeat | 5.27  [4.99-5.55] | 4.38  [4.19-4.57] | 2.45  [2.21-2.69] | 4.98  [4.67-5.29] | 4.19  [3.99-4.39] | 2.39  [2.16-2.62] | 4.85  [4.53-5.17] | 4.14  [3.93-4.35] | 2.33  [2.09-2.57] |
| Entrapment | 5.07  [4.76-5.38] | 4.10  [3.89-4.31] | 1.93  [1.74-2.22] | 4.79  [4.45-5.13] | 3.93  [3.71-4.15] | 2.11  [1.86-2.36] | 4.60  [4.25-4.95] | 3.83  [3.60-4.05] | 1.90  [4.23-4.51] |
| Loneliness | 5.87  [5.75-6.00] | 5.25  [5.16-5.34] | 4.31  [4.17-4.45] | 5.70  [5.57-5.83] | 5.23  [5.13-5.33] | 4.42  [4.28-4.56] | 5.66  [5.51-5.81] | 5.23  [5.13-5.33] | 4.37  [4.23-4.51] |
| Wellbeing | 20.28  [19.87-20.69] | 22.01  [21.72-22.30] | 26.01  [25.37-26.25] | 20.70  [20.26-21.14] | 22.38  [22.07-22.69] | 25.73  [25.29-26.17] | 21.06  [20.56-21.56] | 22.51  [22.19-22.83] | 26.07  [25.63 to 26.51] |

**Table S8: Means and 95% confidence intervals [CIs] of variables by grouping variable over waves (n=3077)**

|  | Wave 1 | | Wave 2 | | Wave 3 | |
| --- | --- | --- | --- | --- | --- | --- |
|  | **Female (n=1692)** | **Male (n=1171)** | **Female (n=1491)** | **Male (n=**1**247)** | **Female (n=** **1389)** | **Male (n=1212)** |
| Defeat | 4.93 [4.74-5.11] | 3.41 [3.21-3.61] | 4.67 [4.47-4.87] | 3.24 [3.04-3.44] | 4.47 [4.26-4.68] | 3.28 [3.07-3.49] |
| Entrapment | 4.62 [4.40-4.83] | 3.14 [2.93-3.35] | 4.40 [4.26-4.66] | 3.03 [2.81-3.25] | 4.16 [3.93-4.39] | 2.95 [2.81-3.11] |
| Loneliness | 5.52 [5.43-5.61] | 4.89 [4.79-4.99] | 5.46 [5.36-5.56] | 4.85 [4.75-4.95] | 5.42 [5.32-5.52] | 4.83 [4.72-4.94] |
| Wellbeing | 21.45 [21.16-21.74] | 23.29 [22.96-23.62] | 22.93 [21.62-22.23] | 23.51 [23.17-23.85] | 22.28 [21.95-22.61] | 23.68 [23.30-24.06] |
|  | **white (n=2777)** | **BAME (n=** **292)** | **white (n=** **2499)** | **BAME (n=236)** | **white (n=2385)** | **BAME (n=213)** |
| Defeat | 4.27 [4.12-4.41] | 4.02 [3.60-4.45] | 4.01 [3.86-4.16] | 4.17 [3.67-4.66] | 3.93 [3.77-4.09] | 3.88 [3.34-4.42] |
| Entrapment | 3.96 [3.80-4.12] | 3.88 [3.39-4.37] | 3.77 [3.60-3.93] | 3.86 [3.34-4.38] | 3.61 [3.44-3.78] | 3.61 [3.06-4.16] |
| Loneliness | 5.23 [5.16-5.30] | 5.36 [5.15-5.57] | 5.16 [5.09-5.23] | 5.40 [5.16- 5.64] | 5.14 [5.06-5.22] | 5.24 [4.99-5.49] |
| Wellbeing | 22.35 [22.12-22.58] | 21.52 [20.81-22.22] | 22.75 [22.51-22.99] | 21.52 [20.69-22.35] | 23.00 [22.74-23.24] | 22.25 [21.25-23.05] |
|  | **High SEG (n=1758)** | **Low SEG (n=1319)** | **High SEG (n=1571)** | **Low SEG (n=1171)** | **High SEG (n=1489)** | **Low SEG (n=1115)** |
| Defeat | 3.83 [3.66-4.00] | 4.81 [4.61-5.01] | 3.64 [3.46–3.81] | 4.53 [4.29–4.77] | 3.60 [3.41-3.79] | 4.35 [4.10-4.60] |
| Entrapment | 3.57 [3.38-3.76] | 4.47 [4.25-4.69] | 3.42 [3.22–3.61] | 4.25 [3.99–4.51] | 3.30 [3.09-3.51] | 4.01 [3.74-4.28] |
| Loneliness | 5.12 [5.03-5.21] | 5.39 [5.30-5.48] | 5.06 [4.96-5.15] | 5.35 [5.24–5.46] | 5.05 [4.96-5.14] | 5.29 [5.16-5.40] |
| Wellbeing | 22.66 [22.38-22.94] | 21.75 [21.45-22.05] | 23.01 [22.91-23.10] | 22.13 [21.76-22.50] | 23.22 [22.91-23.53] | 22.55 [22.17-22.93] |
|  | **Νο ΜΗ (n=2225)** | **ΜΗ (n=852)** | **Νο ΜΗ (n=2002)** | **ΜΗ (n=740)** | **No MH (n=1906)** | **MH (n=698)** |
| Defeat | 3.17 [3.03–3.30] | 7.06 [6.78–7.33] | 3.05 [2.90-3.20] | 6.65 [6.35-6.95] | 2.97 [2.82-3.12] | 6.53 [6.22-6.84] |
| Entrapment | 2.79 [2.64-2.94] | 7.00 [6.68–7.32] | 2.69 [2.54-2.84] | 6.72 [6.37-7.07] | 2.59 [2.43-2.75] | 6.37 [6.01-6.73] |
| Loneliness | 4.84 [4.77-4.91] | 6.28 [6.15-6.40] | 4.83 [4.75-4.91] | 6.15 [6.01-6.29] | 4.83 [4.75-4.91] | 6.02 [5.87-6.17] |
| Wellbeing | 23.66 [23.42-23.90] | 18.64 [18.26-19.02] | 24.01 [23.76-24.26] | 18.94 [18.54-19.35] | 24.18 [23.92-24.44] | 19.50 [19.05-19.95] |

BAME=Black, Asian and minority ethnic, High SEG=ABC1, Low SEG=C2DE, MH=Pre-existing mental health problems

**1. Full details of measures**

**Methods**

Suicidal history was assessed via the following two items adapted from the Adult Psychiatric Morbidity Survey^20^ ‘Have you ever made an attempt to take your life, e.g. by taking an overdose of tablets or in some other way?’ (Yes/No) (suicide attempt history) and ‘Have you ever deliberately harmed yourself in any way but not with the intention of killing yourself?’(Yes/No) (self-harm history). If respondents answered yes to the suicide attempt or the self-harm history questions, they were asked “when was the last time you deliberately harmed yourself?” (past week, past month, past 6 months, more than 6 months, more than 12 months). We report self-harm and suicide attempts in the past week. Suicidal ideation in the last week was assessed by the question ‘How often have you thought about taking your life in the last week? (one day, several days, more than half the days, nearly everyday, never, I would rather not answer)’. Any non-zero response was re-coded as yes to suicidal ideation in the past week. Depression was assessed via the Patient Health Questionnaire (PHQ-9^21^). The GAD-7^22^, a 7-item screening tool, was used to assess generalized anxiety disorder. Both measures ask how often symptoms are bothering the respondents in the last 2 weeks. Scores of 10 and above on both measures are thought to indicate a moderate levels of depression and anxiety and are used as cutoffs here^22,23^. Feelings of defeat (perceived failed struggle and loss of rank) were assessed using four items from Griffiths’ short-form scale^24^. The Entrapment Scale Short-form^25^ was used to explore perceptions of entrapment (feeling trapped by thoughts and feelings or situation). Mental wellbeing was assessed via the 7-item Short Warwick Edinburgh Mental Well-Being Scale (SWEMWBS)^26^. Loneliness was assessed using the UCLA 3-item scale^27^. Four items from the ENRICHD Social Support Instrument (ESSI^28^) were included to assess the availability of social support. The National Readership Survey social grade^17^, derived from the occupation of the main income earner in the household, was used as an indicator of socioeconomic group (SEG): high (A+B+C1) versus low (C2+D+E). To assess pre-existing mental health conditions, participants were first asked if they had any long-standing physical or mental impairment, illness or disability.  Participants were then asked to select their mental or physical impairment from a list of options, which included mental health conditions, neuro-divergent disorders and alcohol and drug problems, and these responses were used to create a dichotomous variable for presence or absence of a pre-existing mental health condition.

**Details of those invited to take part in the study:** the panel has approximately 300,000 registered adult members. In total, 7,471 panel members were invited to take part, 3077 were included in the final sample (target sample was n=3,000) and 4394 did not take part in the survey. The majority was screened out as a particular quota was full (n=3527) and the remainder dropped out (n=867; see supplementary material). 90% of the 4394 of panel members who were invited to participate but did not take part in the survey provided details of their age and gender: 65.6% identified as female and 34.3% as male. The age distribution was as follows: 18-29 years = 19%, 30-59 years = 62.9% and 60+ years =18.1%.”

**2. Breakdown of pre-existing mental health condition (n=3077)**

| Mental health condition or impairment | % (n) |
| --- | --- |
| Depression | 18.0 (555) |
| Anxiety | 21.5 (661) |
| Obsessive compulsive disorder (OCD) | 1.8 (56) |
| Posttraumatic stress disorder (PTSD) | 2.1 (64) |
| Autism or Asperger’s | 1.4 (44) |
| Alcohol or drug problems | 2.2 (68) |
| Other | - 1. (68) |

3. **Differences between participants who completed all waves compared to those who did not on wave 1 measures**

| Variable | Completes all waves (n=2518)  % (n) | Not-complete all waves (n=559)  % (n) | Chi-square (X^2^, df=1) | p |
| --- | --- | --- | --- | --- |
| Suicidal ideation past week | 7.5 (185) | 11.3 (60) | 7.992 | 0.007 |
| PHQ-9 cut-off | 23.9 (602) | 36.0 (201) | 34.435 | <0.0001 |
| GAD-7 cut-off | 18.8 (473) | 30.6 (171) | 38.525 | <0.0001 |
|  | **M (SD)** | **M (SD)** | **t (df=3075)** | **p** |
| Defeat | 4.11 (3.88) | 4.89 (4.10) | -4.26 | <0.0001 |
| Entrapment | 3.80 (4.33) | 4.65 (4.46) | -4.17 | <0.0001 |
| Loneliness | 5.15 (1.91) | 5.65 (1.97) | -5.60 | <0.0001 |
| Wellbeing | 22.56 (6.20) | 20.95 (6.03) | 5.57 | <0.0001 |
